# 18075 Giving birth during COVID-19 from the birthing person’s perspective

**DOI:** 10.1017/cts.2021.604

**Published:** 2021-03-31

**Authors:** Rachel Breman

**Affiliations:** University of Maryland

## Abstract

ABSTRACT IMPACT: This work provides context from the patient perspective on the impact of hospital policies on their birthing experiencing during the first peak of the pandemic. OBJECTIVES/GOALS: The goal of this study was to report the intrapartum care experiences from people giving birth during the COVID-19 pandemic in the United States. Place of birth included hospital, birth center and home births. METHODS/STUDY POPULATION: Studies that involved patient-related data collection are hindered by pandemic-related changes in clinical practices and research policies. Our aim was to assess patient experience during a pandemic, we explored data collection via a large online community of pregnant women. We asked if women who birthed during COVID-19 changed their birth setting and if they experienced less respectful care, more pressure to undergo induction and/or cesarean birth and newborn separation. We also wanted to explore whether there were differences in the care experience depending on the race of the woman. Open ended questions on care experiences were included and content analysis conducted. Bivariate analysis was conducted comparing those from high versus less COVID-19 impacted areas and by race (White/Black self-identifying). RESULTS/ANTICIPATED RESULTS: The mean age was 31.5 years (SD = 5.0), 80.7% identified as White, 85.0% married, and 85.3% privately insured (N=388). Bivariate unadjusted analyses comparing high vs. low impact COVID-19 states, 22.3% considered changing their place of birth versus 12.7% in less impacted areas (p<.05): no difference pressure for induction/cesarean based on region. In bivariate unadjusted analysis comparing White and Black people, Black people had higher odds of pressure for cesarean or induction compared to White (OR 10.3, 95% CI 2.2 to 48.6, p=.0003). Black people had lower respect scores vs. White (68.7 vs. 72.3 p<.01) and higher odds of preterm birth 3.7 (1.1 to 12.4, p=0.04). Content analysis themes were institutional policies, changes in care, hospital staff interactions, sub-par care, support during birth and mental health. DISCUSSION/SIGNIFICANCE OF FINDINGS: The analysis demonstrated differences among participants from highly versus less impacted COVID-19 states. Disparities persisted with Black women reporting lower respect, pressure for interventions and more preterm birth when compared to White participants. Limitations include use of a convenience sample and self-reported data.

